# Eloquence

**Published:** 1858-06

**Authors:** 


### ELOQUENCE.

No man can be eloquent if he speaks laboriously, because, instead of being carried away with the subject, the hearers are in painful sympathy with the speaker. In the estimation of some, eloquence is more a test of physical strength than anything else; it is voice, and nothing more. It is the earnest, but undemonstrative manner, which carries away the hearer. Who ever heard of a judge pronouncing sentence of death with stamping feet, with meaningless gesture, and with thundering vociferation? It is the soft utterance of irrepressible emotion which brings tears to the eyes of those who seldom weep.

A man who feels deeply, can speak an hour, without appreciated effort; while, in the same time, the heartless vociferator will be bathed in perspiration. Many a case of incurable *Throat Ail* has resulted from boisterous speaking. Let all those, then, whose "whole stock in trade" is their voice, learn this practical lesson. Be deeply impressed with the importance of your subject, let your whole heart be in it; imbue yourself with a full sense of your high responsibility, and nature and instinct will suggest a mode and manner which will well nigh be resistless; and you may speak a full hour unwearied.

